# Delirium-Induced Takotsubo Cardiomyopathy

**DOI:** 10.7759/cureus.37941

**Published:** 2023-04-21

**Authors:** Olawole Akinboboye, Sheri Walls

**Affiliations:** 1 Internal Medicine, Piedmont Athens Regional Medical Center, Athens, USA

**Keywords:** echo cardiogram, apical akinesia, toxic delirium, delirium, takotsubo cardioyopathy

## Abstract

An 85-year-old woman presented with altered mental status and appeared to be actively agitated due to her medications. During her hospitalization, troponins trended up and an electrocardiogram (ECG) showed diffuse ST elevation. Echocardiogram showed an estimated ejection fraction of 40% with hypokinesis of the apex, suggestive of Takotsubo cardiomyopathy. After several days of supportive care, the patient showed significant clinical improvement with normalization of ECG, cardiac enzymes, and echocardiographic findings. Although Takotsubo cardiomyopathy has been associated with diverse forms of physical or emotional stress, this report discusses a rare case of delirium state causing Takotsubo cardiomyopathy.

## Introduction

Takotsubo cardiomyopathy, also known as stress-induced cardiomyopathy, is a clinical syndrome characterized by transient acute apical ventricular dysfunction in the absence of significant obstructive coronary artery disease; it is usually triggered by physical or emotional stressors [1]. Its clinical manifestations mimic that of acute coronary syndrome with typical ST-T wave changes on electrocardiogram (ECG) and elevated cardiac enzymes [1]. In this case report, we present a patient with Takotsubo cardiomyopathy induced by delirium.

## Case presentation

An 85-year-old Caucasian woman with a medical history of heart failure with preserved ejection fraction, obstructive sleep apnea, chronic back pain, and thyroid disease presented to the emergency room after her family called the emergency medical service with a complaint of altered mental status. The patient’s husband reported that she had taken two tramadol pills a few minutes prior to her presentation. The patient reported that there were no additional medications. Upon arrival at the emergency department, her initial vitals were as follows - temperature: 97.2 °F, pulse rate: 101 beats per minute, respiratory rate: 22 breaths per minute, blood pressure: 175/83 mmHg, and oxygen saturation: 96% on room air. On the physical exam, the patient was extremely agitated, yelling that someone was trying to harm her, and aggressively flailing her hands and legs, and she required four-point restraints. ECG done initially on presentation showed normal sinus rhythm with the previous right bundle branch block. Laboratory examination initially revealed serum creatinine of 1.26 mg/dL. Urinalysis revealed few bacteria with 9 WBC and 2+ leukocytes, and urine culture showed 10,000-50,000 Enterococcus faecalis (which was treated during the hospitalization course). Alcohol tests, urinary drug screening, and lactate were within normal limits. Chest X-ray and CT head were unremarkable. Her initial troponin I levels were 13 ng/L and 14 ng/L (normal range: 0-15 ng/L) for the zero-hour and two-hour checks, respectively; however, the six-hour check was elevated at 181 ng/L. The patient still was unable to follow commands and respond at this time, and hence, given the elevated troponin levels, a repeat test was done, which showed a troponin level of 7900. At this time, the patient still appeared to be in no acute distress, but due to this troponin finding, an ECG was done, which showed ST elevation in the anterior leads. The patient was started on a heparin drip and then cardiology was consulted. An echocardiogram was performed (Video 1), which showed Takotsubo cardiomyopathy, hypokinesis at the apex, and a left ventricle systolic function of 41%.

**Video 1:** Echocardiogram long-axis view showing Takotsubo cardiomyopathy

Following this, cardiology reported that the initial plan was to investigate further with coronary angiography to rule out a myocardial infarction as per the required criteria to confirm normal coronary arteries or minimal coronary artery disease; however, due to the recent combative behavior and denial of any complaints, they proposed continuing with the heparin drip for 48 hours and her beta blocker. During the course of admission, her troponin levels downtrended back to normal limits, her mental status improved, and she was eventually found to be oriented to time, place, and person. The plan on discharge was for the patient to follow up in the clinic with cardiology to repeat an echocardiogram; however, she has been lost to follow-up so far.

## Discussion

Takotsubo cardiomyopathy was first reported in Japan in the 1990s and did not emerge in the United States until almost the early 2000s. The condition is often referred to as transient apical ballooning syndrome, broken heart syndrome, ampulla cardiomyopathy, or stress-induced cardiomyopathy [1]. As its name indicates, this phenomenon is often triggered by stressful events such as emotional or physical trauma, especially among women over the age of 50 years. Other causes include the loss of a loved one, natural disasters, fierce arguments, surgery, drug withdrawals, and even relationship conflicts [1,2]. Usually, the patients do not have any of the major cardiovascular risk factors such as diabetes, hypertension, hyperlipidemia, alcohol use disorder, and/or tobacco use disorder [3].

This acute illness is a benign condition; however, it is associated with several etiological and pathophysiological phenomena. The most common one involves the incidence of a stressful event, which causes our bodies to go into a “fight or flight” mode that causes a catecholamine surge [4]. This catecholamine surge involves a profound release of norepinephrine and epinephrine that causes significant stress on the heart, which may lead to patients feeling that they are on the verge of a heart attack. Patients may present with acute-onset chest pain that requires the initial workup in order to rule in or out cardiopulmonary and/or vascular causes [1,3]. This workup includes ECG, chest X-ray, and troponins. ECG during this transient process may reveal ST-segment elevations and elevated troponin levels. If the patient then undergoes a cardiac catheterization, it may reveal left ventricular apical ballooning in the absence of any evidence of coronary artery disease. The modified Mayo criteria have provided clinicians with the tools to diagnose this transient illness [2]. The manifestations include the absence of coronary artery disease on cardiac angiography, ECG evidence of ST-segment elevation and/or T wave inversion, the modest elevation of troponin levels, absence of myocarditis or pheochromocytoma, transient dyskinesia, hypokinesis, or akinesis of the left ventricle [2]. Our patient had a significant elevation of her troponin, apical dyskinesia appreciated on the echocardiogram, and ST elevation in the anterior leads, which makes Takotsubo the most likely diagnosis. However, the patient has yet to follow up with cardiology for repeat imaging. 

The prognosis in these patients is favorable as general symptomatic and supportive measures lead to the left ventricle systolic function usually returning to normal within a few weeks [5]. However, unstable patients should be managed in high-dependency or intensive care units with mechanical support (intra-aortic balloon counterpulsation, bridge to recovery treatment with extracorporeal membrane oxygenation, temporary left ventricular assist devices) provided to patients with low cardiac output and cardiogenic shock [6,7].

## Conclusions

Stress cardiomyopathy is a transient phenomenon mostly seen in post-menopausal women. It causes dysfunction of the left ventricle, which can mimic several cardiopulmonary processes in the absence of coronary artery disease. It can be caused by multiple factors, and it appears that it can be precipitated by delirium in rare cases; this association has not been described in the literature. We hope this case report will encourage physicians to have a broader differential when encountering patients who present with delirium-related pain, which would lead to a prompt diagnosis and appropriate management of this transient condition.
